# The global distribution of CO_2_-rich magmas is determined by lithospheric thickness

**DOI:** 10.1038/s41561-026-01990-7

**Published:** 2026-05-22

**Authors:** Emilie E. Bowman, Sally A. Gibson, Siyuan Sui, Sergei Lebedev

**Affiliations:** Department of Earth Sciences, University of Cambridge, Cambridge, UK

**Keywords:** Petrology, Geochemistry

## Abstract

Metasomatized lithospheric mantle plays a critical role in the petrogenesis of CO_2_-rich magmas, which are important hosts of rare-earth element deposits. However, the relationship between the structure of the lithosphere and the global distribution of CO_2_-rich magmas remains poorly quantified. Here we analyse the locations of young (<200 million years ago) continental intraplate CO_2_-rich silicate magmas and magmatic carbonatites in conjunction with upper-mantle shear-wave velocity anomalies and lithospheric thickness estimates. Our results document systematic increases in lithospheric thickness with estimated magma CO_2_ content from basanites (<5 wt% CO_2_), which erupt through seismically slow and thin non-cratonic lithosphere, to nephelinites, melilitites and ultramafic lamprophyres, which occur within progressively faster, thicker lithosphere and, finally, to lamproites and kimberlites (<20 wt% CO_2_), which are emplaced on thick cratonic lithosphere. Carbonatites are associated with lithospheric thicknesses similar to those of nephelinites, melilitites and ultramafic lamprophyres, implying the derivation of carbonatites from these mafic CO_2_-rich silicate magmas via liquid immiscibility and/or fractional crystallization. We illustrate our lithospheric thickness–magma type relationship using Cretaceous–Pleistocene alkaline magmatism across western North America, ultimately demonstrating how lithospheric thickness controls the global occurrence of CO_2_-rich magmas and, consequently, their associated rare-earth element deposits.

## Abstract (web summary)

Continental lithosphere thickness exerts a positive control on CO_2_ contents of erupted magmas, hence also influencing the location of rare-earth element deposits, according to a study of lithospheric thickness and CO_2_-rich magma locations.

## Main

CO_2_-rich primary magmas, although often confined to small-volume systems within intraplate regions, are major sources of atmospheric CO_2_, diamonds and the rare-earth and high-field strength elements^1–4^ that are relied upon globally to achieve energy decarbonization^5^. An understanding of where these magmas are emplaced on the surface of the Earth and the processes that control their spatial distribution is thus crucial to advancing our knowledge of global CO_2_ cycling as well as the locations of economic mineral deposits.

Previous studies have observed that basanites (<5 wt% CO_2_)^6^ primarily occur in off-craton settings characterized by thin lithosphere (<100 km)^7^. In comparison, kimberlites (up to 20 wt% CO_2_)^8^, the primary hosts of diamond deposits, are preferentially emplaced within the interiors of cratons^9,10^, defined herein as regions of >160-km-thick lithosphere that have experienced >2.5 Gyr of tectonic stability^11^. Carbonatites (>25 wt% CO_2_)^12^, which are associated with economic deposits of phosphate, fluorite, Nb, Ta, Zr and the rare-earth elements (REEs)^13–15^, are often concentrated along the steep edges of cratons^4,11^. There is thus evidence to suggest that the global architecture of the lithosphere exerts a first-order control on the emplacement locations of our planet’s most CO_2_-rich magmas. This relationship is expected, as the lower lithosphere has been shown to be a locus of persistent metasomatic enrichment and CO_2_ accumulation driven by the infiltration of small-fraction mantle melts rich in volatiles and incompatible elements^3,16^.

However, despite the well-constrained spatial association between cratonic lithosphere and the emplacement locations of basanites, kimberlites and carbonatites, existing lithospheric thickness estimates for these lithologies are only semiquantitative: basanites are associated with <100-km-thick lithosphere^7^; kimberlites, >140 km (refs. ^9,10^); and carbonatites, >100 km (ref. ^11^). Furthermore, estimates of lithospheric thickness associated with these magma types derive from varying inversions of seismic data, thus rendering direct comparisons of lithospheric thickness among these magma types unreliable. In addition, the relationship between lithospheric thickness and the distribution of other CO_2_-rich magmas—such as lamproites, ultramafic lamprophyres, melilitites and nephelinites—remains unquantified globally. Here, by analysing the locations of these magmas and their compositions in relation to global shear-wave velocity anomalies and internally consistent estimates of lithospheric thickness, we show how the thickness of the lithosphere determines the generation of the full spectrum of carbonated (<5 to >25 wt% CO_2_) magmas.

## Lithospheric controls on CO_2_-rich silicate magmas

**Fig. 1:**
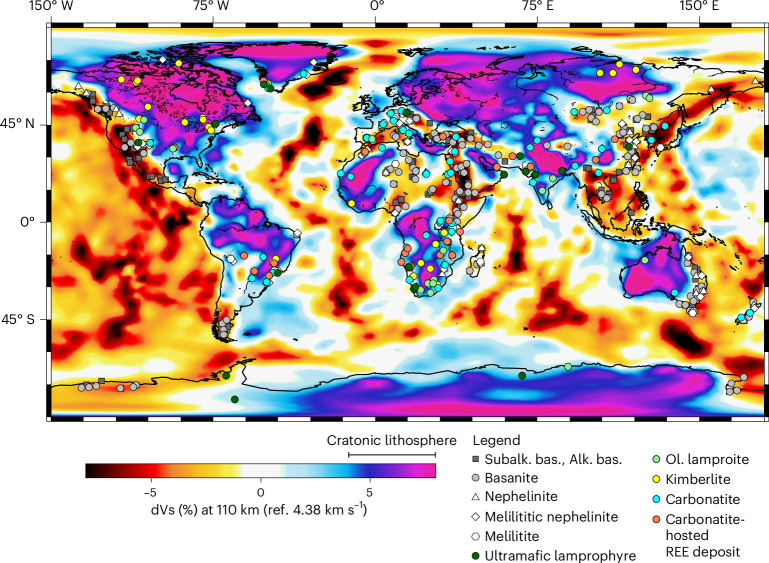
Global shear-wave velocity anomalies (dVs) at 110-km depth showing emplacement locations of young (<200 Ma) continental intraplate CO_2_-rich magmas. Samples have been spatially averaged into 4° × 4° bins for clarity. Cratonic lithosphere is characterized by velocity anomalies >4% above the reference value (4.38 km s^−1^). Data sources for samples are listed in Methods and Supplementary Data 1. Shear-wave velocity anomalies are from the SL2013 tomographic model^17^. Alk. bas., alkali basalt; Ol. lamproite, olivine lamproite; REE, rare-earth element; Subalk. bas., subalkaline basalt.

**Fig. 2:**
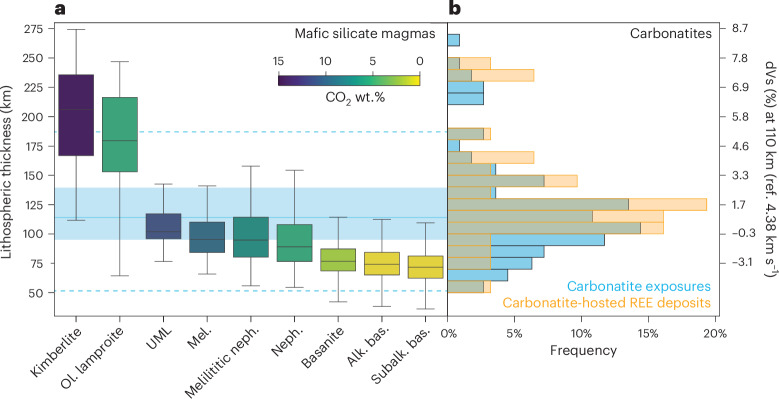
Lithospheric thickness estimates and seismic velocity anomalies for carbonated silicate magmas, carbonatites and carbonatite-hosted REE deposits. **a**, Box-and-whisker plots showing covariations between mafic (>5 wt% MgO) silicate magmas, lithospheric thickness (left-side *y* axis) and seismic velocity anomalies (dVs) at 110 km (right-side *y* axis). Boxes are coloured according to median CO_2_ content of each magma type, calculated using the SiO_2_–CO_2_ parameterization of Dasgupta et al.^38^. Box-and-whisker plot lines correspond to median lithospheric thickness estimates, boxes demarcate the 25th and 75th percentiles (interquartile range, IQR) of the data, and whiskers represent the minimum and maximum values excluding outliers (>1.5 × IQR). Dashed blue lines (whiskers), the blue box (IQR) and the solid blue line (median) correspond to the box-and-whisker plot of carbonatites. Abbreviations and sample sizes (*n*) are as follows: kimberlite (*n* = 52), olivine lamproite (Ol. lamproite, *n* = 35), ultramafic lamprophyre (UML, *n* = 22); melilitite (Mel., *n* = 42); melilititic nephelinite (Melilititic neph., *n* = 97), nephelinite (Neph., *n* = 117), basanite (*n* = 312), alkali basalt (Alk. bas., *n* = 358); subalkaline basalt (Subalk. bas., *n* = 297). **b**, Frequency histogram showing lithospheric thickness (left-side *y* axis) and dVs values at 110 km (right-side *y* axis) for carbonatites (blue) and associated REE deposits (orange). For both plots, dVs at 110 km is calculated from lithospheric thickness estimates according to the dVs–lithospheric thickness correlation shown in Extended Data Fig. 2. Data sources for samples in both **a** and **b** are listed in Methods and Supplementary Data 1.

We build a global dataset of young (<200 million years ago (Ma)) continental intraplate carbonatites and CO_2_-rich mafic (>5 wt% MgO) silicate igneous rocks, which we separate into magma type (kimberlite, olivine lamproite, ultramafic lamprophyre, melilitite, melilititic nephelinite, nephelinite, basanite; Supplementary Data 1). Samples from each magma type were grouped into 1° × 1° bins to reduce bias from densely sampled regions. We then correlated this spatially averaged dataset with seismic shear-wave velocity anomalies at 110 km from the SL2013 tomographic model^17^ (Fig. 1) as well as lithospheric thickness estimates for each binned sample (Fig. 2) calculated using thermodynamic inversion of seismic data^18^ (see Methods for details). Lithospheric thicknesses computed for each magma type are reported in Extended Data Table 1 and Supplementary Data 1.

**Extended Data Fig. 1:**
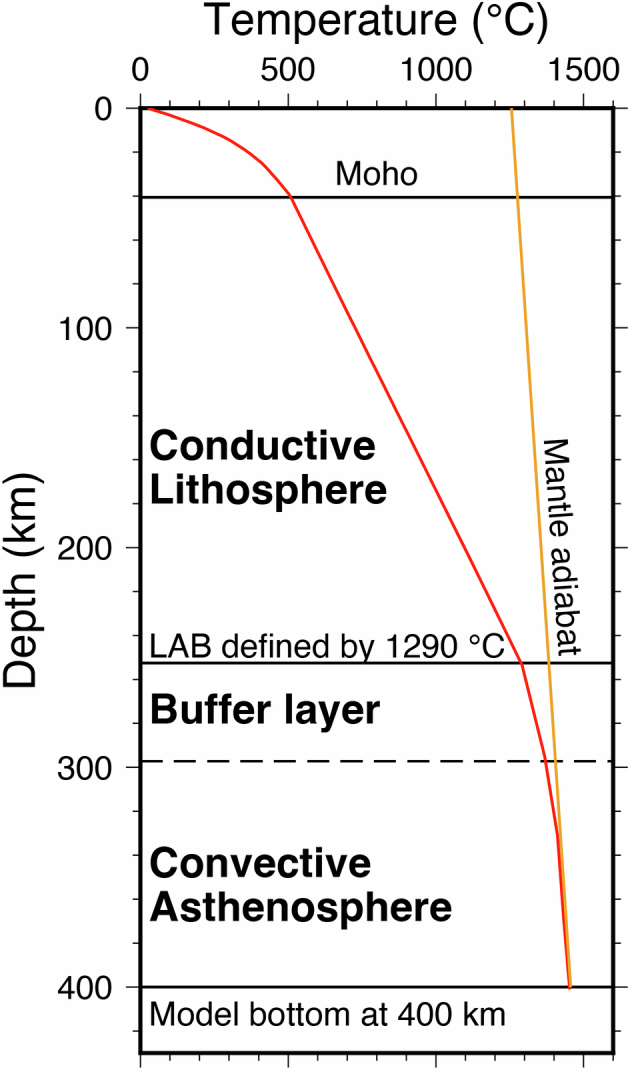
Schematic diagram of an example temperature profile and our definition of the lithosphere-asthenosphere boundary (LAB). The red line represents the temperature profile and the yellow line represents the mantle adiabat.

**Extended Data Fig. 2:**
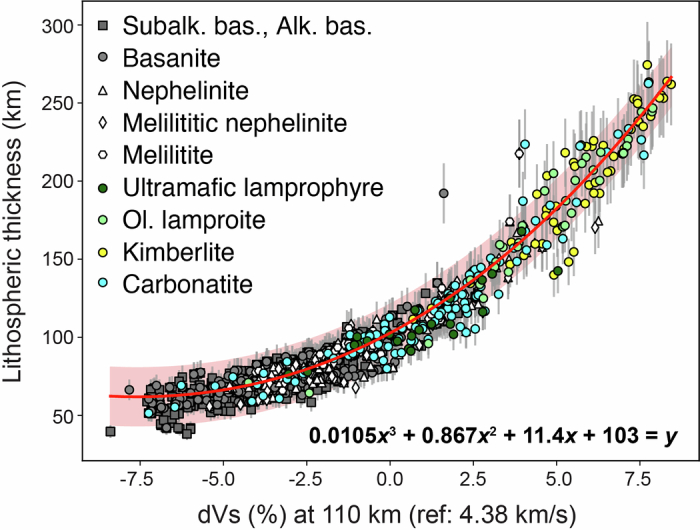
Covariation between shear-wave velocity anomalies (dVs) at 110 km and lithospheric thickness estimates for our spatially averaged magma type dataset. Shear-wave velocity anomalies at 110 km are from Schaeffer and Lebedev^17^. Gray error bars define a 10% uncertainty on each median lithospheric thickness estimate. The red curve is a weighted best-fit line through the correlation (n = 1443), while the red envelope represents the 2-sigma standard deviation on the best-fit line. Abbreviations as in Fig. 1. Data sources for samples are listed in Methods and Supplementary Data 1.

Although compositional heterogeneity in the mantle can produce seismic shear-wave velocity perturbations of up to ~1%^19^, shear-wave velocity profiles through the mantle lithosphere predominantly reflect variations in temperature^18^. Lithospheric temperature, in turn, depends on the depth of the lithosphere–asthenosphere boundary (LAB)^20,21^, here defined as the 1,290 °C isotherm that separates the fully conducting mechanical lithosphere from the convecting mantle (Extended Data Fig. 1). As a result, because shear-wave velocity anomalies (dVs) determined for lithospheric depths (for example, 110 km) represent weighted averages around the depth range of the LAB, these anomalies can be used as proxies for global variations in lithospheric architecture: large positive deviations (>4%) in shear-wave velocity from the global average (4.38 km s^−1^) characterize cold and thick cratonic lithosphere, whereas lower dVs values signal the presence of warmer and thinner lithosphere^20,22^. Because the seismic velocity anomaly for each binned sample in our dataset increases monotonically with increasing lithospheric thickness (Extended Data Fig. 2), we use these datasets in tandem to quantify the relationship between the structure of the lithosphere and the global distribution of CO_2_-rich magmas.

**Extended Data Fig. 3:**
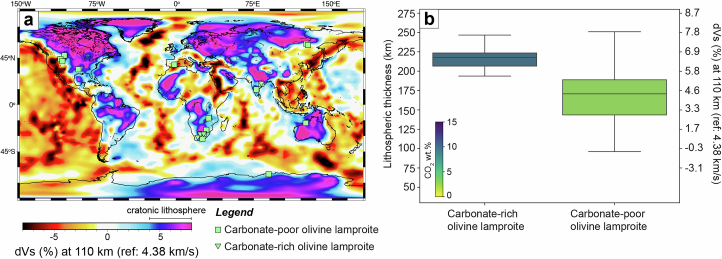
Seismic shear-wave velocity anomalies and lithospheric thickness estimates for carbonate-rich and carbonate-poor olivine lamproites. **a**, Seismic shear-wave velocity anomaly map at 110 km (ref. ^17^) showing emplacement locations of young ( < 200 Ma) continental intraplate carbonate-rich and carbonate-poor olivine lamproites. Except for one locality in India, carbonate-rich olivine lamproites are confined to South Africa. **b**, Box-and-whisker plot showing the lithospheric thickness of carbonate-rich (n = 16) and carbonate-poor (n = 27) olivine lamproites. The majority of carbonate-rich olivine lamproites are located within thicker lithosphere (205-225 km) compared to carbonate-poor lamproites (145-190 km). Box-and-whisker plot lines correspond to median lithospheric thickness estimates, boxes demarcate the 25^th^ and 75^th^ percentiles (interquartile range; IQR) of the data, and whiskers represent the minimum and maximum values excluding outliers ( > 1.5*IQR). Data sources for samples in both (**a**) and (**b**) are listed in Methods and Supplementary Data 1.

**Extended Data Fig. 4:**
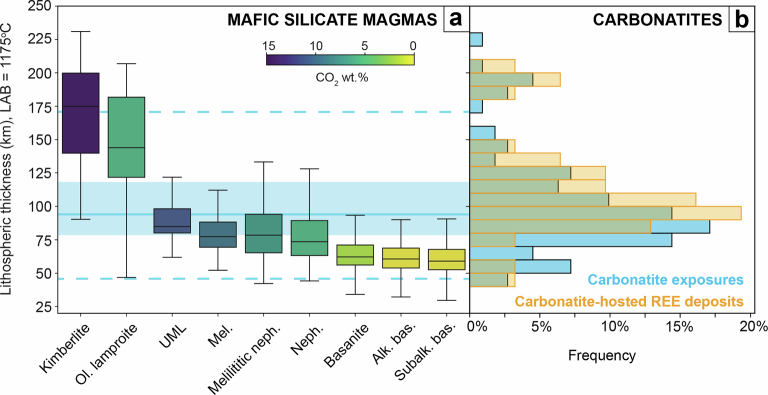
Lithospheric thickness estimates for carbonated silicate magmas, carbonatites, and carbonatite-hosted REE deposits calculated using an LAB temperature of 1175 °C. **a**, Box-and-whisker plots showing covariations between mafic ( > 5 wt.% MgO) silicate magmas and lithospheric thickness. Boxes are coloured according to median CO_2_ content of each magma type, calculated using the SiO_2_-CO_2_ parameterization of Dasgupta et al.^38^. Box-and-whisker plot lines correspond to median lithospheric thickness estimates, boxes demarcate the 25^th^ and 75^th^ percentiles (interquartile range; IQR) of the data, and whiskers represent the minimum and maximum values excluding outliers ( > 1.5*IQR). Blue dashed lines (whiskers), the blue box (IQR), and the solid blue line (median) correspond to the box-and-whisker plot of carbonatites. Abbreviations and samples sizes (n) are as followed: Kimberlite (n = 52), olivine lamproite (Ol. lamproite; n = 35), ultramafic lamprophyre (UML, n = 22); melilitite (Mel., n = 42); melilititic nephelinite (Melilititic neph., n = 97), nephelinite (Neph., n = 114), basanite (n = 303), alkali basalt (Alk. bas., n = 335); subalkaline basalt (Subalk. bas., n = 269). **b**, Frequency histogram showing lithospheric thickness for carbonatites (blue) and associated REE deposits (orange). Data sources for samples in both (**a**) and (**b**) are listed in Methods and Supplementary Data 1.

**Extended Data Fig. 5:**
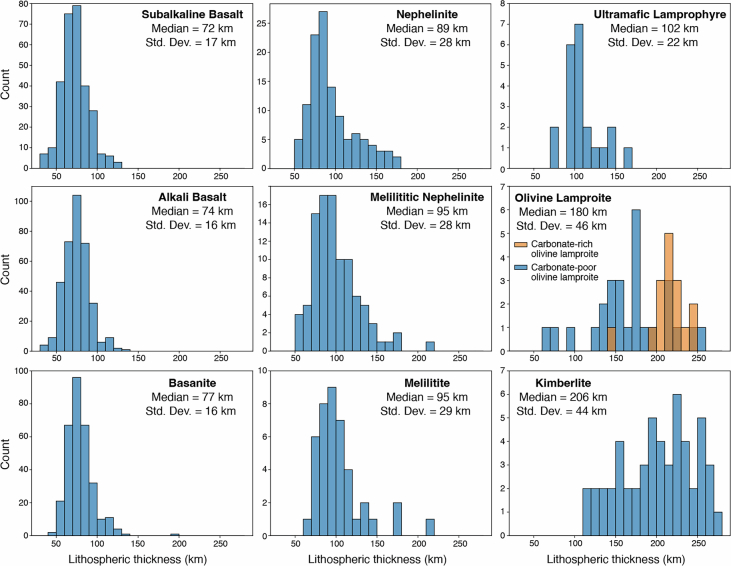
Histograms showing lithospheric thickness estimates for the full spectrum of CO_2_-rich mafic silicate magmas. For each magma type, the median lithospheric thickness and 1-sigma standard deviation (Std. Dev.) are reported. Sample size information as in Fig. 2. Data sources for samples are listed in Methods and Supplementary Data 1.

We find that distinct systematic covariations exist between young (<200 Ma) CO_2_-rich mafic silicate magmas and the seismic velocity and lithospheric thickness of the region within which the majority of each magma type are emplaced (Figs. 1 and 2a). Kimberlites, for example, are emplaced preferentially within cratonic cores (Fig. 1) where velocity anomalies are high (4.4–7.3% dVs; Fig. 2a) and the lithosphere is thick (170–235 km), a finding emphasized by previous studies^9,10^. Olivine lamproites, in comparison, predominantly occur within cratonic to pericratonic regions characterized by slightly lower dVs (3.6–6.5%) and thinner lithosphere (155–215 km; Figs. 1 and 2a and Extended Data Fig. 3). By contrast, ultramafic lamprophyres are typically emplaced on top of 95–120-km-thick (−0.7% to 1.4% dVs) lithosphere (Figs. 1 and 2a). Even lower median seismic velocity anomalies (−2.4% to 1.0%) and lithospheric thicknesses (80–115 km) characterize the spatial distribution of melilitites, melilititic nephelinites and nephelinites (Figs. 1 and 2a). These rocks, although observed in some regions (for example, southern Africa and eastern South America) to occur just beyond the margins of cratons, primarily erupt in areas distal to thick craton interiors. Basanites, alkali basalts and subalkaline basalts, consistent with the results of Ball et al.^7^, are associated with the lowest median velocity anomalies (−7.5% to −1.3%; Fig. 2a) and thinnest lithosphere (60–90 km) and erupt along prominent bands of low shear-wave velocities in off-craton continental settings (Fig. 1). Importantly, lithospheric thickness increases systematically from basalts to kimberlites even when the LAB is defined as a colder isotherm (for example, 1,175 °C; Extended Data Fig. 4), thus demonstrating that the observed lithospheric thickness–magma type relationship persists despite the chosen definition of the LAB. Finally, we note that post-emplacement changes in lithospheric thickness cannot be completely ruled out; however, because the median lithospheric thickness associated with the full spectrum of CO_2_-rich magmas exhibits a robust increase from basalts to kimberlites, any such post-emplacement changes are likely to contribute only to scatter in the data (Fig. 2 and Extended Data Figs. 2 and 5).

**Fig. 3:**
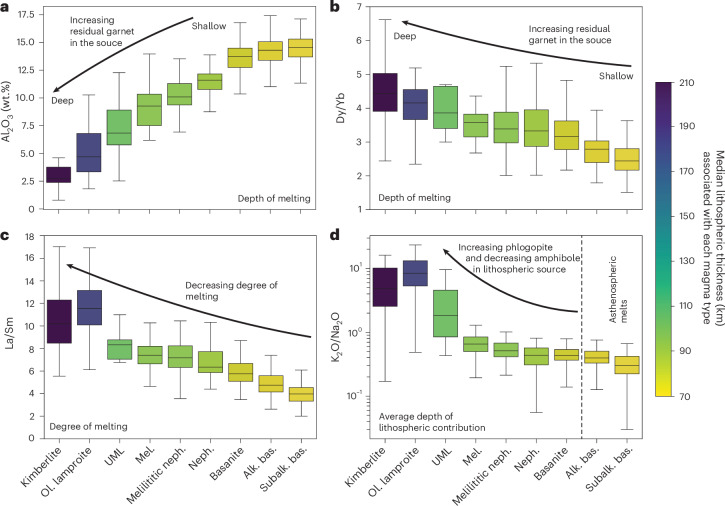
Geochemistry of CO_2_-rich silicate magmas. **a**–**d**, Box-and-whisker plots showing Al_2_O_3_ (**a**), Dy/Yb (**b**), La/Sm (**c**) and K_2_O/Na_2_O (**d**) of primitive (> 8 wt% MgO) CO_2_-rich silicate magmas coloured according to the median lithospheric thickness of each magma type. Box-and-whisker plot lines correspond to the median value of the geochemical parameter, boxes demarcate the 25th and 75th percentiles (IQR) of the data and whiskers represent the minimum and maximum values excluding outliers (> 1.5 × IQR). Abbreviations as in Fig. 2. Sample sizes for each box-and-whisker plot are reported in Extended Data Table 2. Geochemical data sources are listed in Methods and Supplementary Data 1.

Our lithospheric thickness estimates are important in that they represent the maximum depth of lithospheric melting for each magma type, which may also include contributions from the sublithospheric convecting mantle^23^. In fact, these magma type-dependent variations in lithospheric thickness are mirrored by changes in major and trace element proxies sensitive to depth of melting within our spectrum of CO_2_-rich silicate magmas (Extended Data Table 2), which we further filter to include only primitive samples with MgO >8 wt% that have primarily undergone only olivine fractionation. Median Al_2_O_3_ contents and Dy/Yb ratios, for example, progressively decrease and increase, respectively, from basanites to kimberlites (Fig. 3a,b). To a first order, these trends signal greater pressures of melting within the garnet stability field as lithospheric thickness increases^24,25^, consistent with results from partial melting experiments on alkali- and volatile-rich lithologies^26^. According to experimental results, basanites, nephelinites and melilitites can form via partial melting of amphibole-rich lithologies at the relatively low pressure of 1.5 GPa (50 km)^27,28^. Basanitic melts can also derive from partial melting of pyroxenitic lithologies at pressures of 2–2.5 GPa (65–80 km)^29^. In comparison, nephelinites and melilitites can be generated at even greater pressures (2.5–3 GPa; 80–100 km) from incipient melting of alkali- and volatile-rich peridotitic compositions^30–33^. From these source lithologies, pressures of ≥4 GPa (≥130 km depths) are necessary to produce melts of ultramafic lamprophyric composition^33,34^, whereas olivine lamproites require higher pressures (≥5 GPa; ≥165-km depths) to form^35^. Finally, kimberlites, the deepest derived melts on Earth, have been shown to form from carbonated peridotite at pressures >6 GPa (refs. ^33,36,37^).

Our results further display a systematic increase in median La/Sm from basanites to kimberlites, indicating smaller degrees of melting (or greater source enrichment) with increasing lithospheric thickness (Fig. 3c). These trends are mirrored by variations in the CO_2_ contents of each magma type (Fig. 2a), which we calculate according to the SiO_2_–CO_2_ parameterization of experimental data by Dasgupta et al.^38^. With the exception of olivine lamproites, which have relatively high H_2_O/CO_2_ ratios^39^, median CO_2_ contents systematically rise with lithospheric thickness, as CO_2_ is increasingly concentrated in progressively deeper, lower-degree melts in accordance with the incompatible behaviour of carbon during mantle melting.

Finally, we observe an increase in the median K_2_O/Na_2_O ratio from basanites to kimberlites (Fig. 3d). Specifically, the compositions of CO_2_-rich silicate magmas shift from sodic (K_2_O/Na_2_O of <1; basanite through melilitite) to potassic (K_2_O/Na_2_O of >1; ultramafic lamprophyre through kimberlite) as lithospheric thickness increases. This shift marks the destabilization of pargasite at 80–120 km depth, allowing phlogopite ± K-richterite to become the dominant melt-producing hydrous phases in the deeper metasomatized lithosphere^40–42^. This compositional shift thus reflects deepening of the lithospheric contribution to CO_2_-rich silicate magmatism as the lithosphere thickens.

Overall, our results support a scenario in which the full spectrum of CO_2_-rich mafic silicate magmas, from kimberlites to basanites, is generated in regions underlain by progressively thinner lithosphere, a pattern that exists regardless of the definition of the mechanical LAB. Such a pattern of magmatism may occur during continental rifting or mantle plume activity, where 20 Myr of lateral heat delivery to the margins of cratons, potentially accompanied by convective removal of lithospheric material, leads to tens of kilometres of lithospheric thinning and/or vertical heat conduction as well as partial melting of the metasomatized lithospheric mantle^11,43–45^. This model is consistent with apparent temporal variations in melt composition during continental rifting within individual magmatic provinces, for example, along the margins of the Labrador Sea^46^.

## Lithospheric controls on carbonatites

Our results also provide insight into the primary origin of carbonatites and their associated REE deposits. Decades of research has elucidated three main processes that are directly responsible for carbonatite petrogenesis: (1) partial melting of carbonated lithospheric and/or asthenospheric mantle^30,33,47^, (2) fractional crystallization of CO_2_-rich silicate magmas^48,49^ and (3) carbonatite unmixing from CO_2_-rich silicate magmas^49–52^. For carbonatite magmas to be generated in the asthenospheric mantle, partial melting of carbonated peridotite must occur at pressures >6 GPa to achieve degrees of melting low enough to generate a melt sufficiently enriched in CO_2_ (ref. ^53^). However, our results show that carbonatite localities are typically associated with lithospheric thicknesses ranging between 95 and 140 km (−1% and 2.6%), with a median of 114 km (Fig. 2). Such shallow LAB depths (equivalent to ~3–4.5 GPa) allow partial melting at lower pressures and higher melt fractions within the asthenosphere, thus diluting melt CO_2_ contents and generating carbonated silicate magmas in lieu of carbonatites^11^. Our results therefore preclude a direct origin of the majority of carbonatites from the convecting asthenospheric mantle. While our analysis cannot rule out partial melting of the lithospheric mantle as a primary origin of carbonatite magmas, these small-volume reactive melts, as evidenced by carbonated mantle xenoliths^54^, are typically consumed as they interact with the lithospheric mantle and are thus unlikely to reach the surface^55^.

An important finding from our work is that most carbonatites, including those associated with REE deposits, are located where seismic velocity and lithospheric thickness ranges are similar to those of the CO_2_-rich ultramafic lamprophyres, melilitites, melilititic nephelinites and nephelinites (Fig. 2). Distinct tails in the frequency distribution of carbonatite occurrences and REE deposits also overlap the lithospheric thickness ranges of kimberlites and basanites (Fig. 2b). These findings support the generation of most carbonatites through liquid immiscibility and/or crystal fractionation from CO_2_-rich silicate magmas, particularly nephelinites, melilitites and ultramafic lamprophyres^48–52^, which occurs in the crust as the parental melt cools and crystallizes. The similarity in the spatial distribution of carbonatites and their associated REE deposits (Fig. 2b) indicates that it is most likely secondary processes, such as crystal fractionation or hydrothermal alteration^56^, that trigger economic mineralization of carbonatitic intrusions.

## Application to North America

**Fig. 4:**
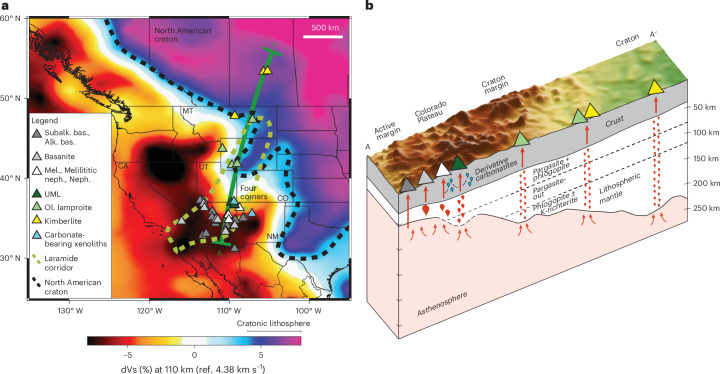
Location of CO_2_-rich magmas in North America with respect to seismic shear-wave velocity anomalies and lithospheric thickness. **a**, Shear-wave seismic velocity anomaly map^17^ of western North America depicting the distribution of Cretaceous to Pleistocene CO_2_-rich magmas and carbonate-bearing xenoliths^61,62^ along transect A–A′, which is shown in **b**. The locations of the Laramide corridor^58,59^ and the North American craton are also shown as dashed green and black lines, respectively. Data sources for samples are listed in Methods and Supplementary Data 1. **b**, Idealized cross section along transect A–A′ showing occurrences of CO_2_-rich magmas with respect to the configuration of the continental lithospheric mantle. Lithospheric thicknesses are computed for the transect line A–A′ (see Methods for details) using an interval of 100 km between the two end points. As the relatively small block of thick lithosphere beneath the Colorado Plateau is not resolvable accurately in the smooth global tomography, we use lithospheric thickness estimates from receiver functions^64–66^ for this narrow feature (dashed LAB). The relative size of silicate melt droplets (red droplets) indicates the degree of melting. The location of silicate melt droplets represents the depths at which the lithosphere may be melted by each magma type, with the average depth of lithospheric contribution increasing from basanites to kimberlites. Red arrows below the LAB indicate potential sublithospheric contributions to carbonated silicate magmatism. Carbonatites form in the crust (green melt droplets) through fractional crystallization and/or liquid immiscibility from melilitite through nephelinite and ultramafic lamprophyre parental magmas. The depth range of pargasite-out (80–120 km) is highlighted^40,42^. Crustal thickness is from CRUST1.0^67^. Topographic data are from SRTM15+^68,69^, filtered and plotted by the PyGMT Python wrapper for GMT^70,71^. Abbreviations as in Fig. 2.

The lithospheric thickness–magma type relationship described above is exemplified along a transect from the North American craton into the Laramide corridor of western North America (Fig. 4). The high-velocity North American craton in Canada is host to Cretaceous diamondiferous kimberlites sourced in part from the thick, heavily metasomatized cratonic lithosphere^57^. To the south, the Laramide corridor, extending from the low-velocity active margin of southern California to the western extent of the North American craton in Montana, marks the region affected by low-angle subduction of the Farallon slab from 88 to 68 Ma (refs. ^58,59^). Devolatization of the slab led to metasomatism of the overriding continental lithosphere^60^. Subsequent rollback and detachment of the subducting slab and later continental extension drove partial melting of this metasomatized lithosphere during the Eocene–Pleistocene, leading to the eruption of CO_2_-rich magmas throughout western North America.

Along this transect, changes in the geochemistry of CO_2_-rich magmas coincide with an increase in seismic velocity and lithospheric thickness as the North American craton is approached from the west (Fig. 4). Specifically, we observe that: (1) basanites occur preferentially in regions of slow, thin lithosphere along the active margin of western North America; (2) nephelinites, melilitites and ultramafic lamprophyres are emplaced through lithosphere of intermediate velocity and thickness closer to the North American craton; and (3) lamproites and kimberlites are found along the margins of the North American craton, the latter of which extend into the seismically faster, thicker lithosphere within the Canadian interior of the North American craton. While bona fide carbonatites have not been identified along this transect, mantle xenoliths exhumed during the Neogene in the Four Corners region—host to abundant ultramafic lamprophyres together with melilitites and nephelinites—contain primary carbonates interpreted to derive from carbonatite melts^61,62^. According to our results, these melts most likely formed as derivative liquids from parental CO_2_-rich silicate magmas (Fig. 4).

Thus, our quantified lithospheric thickness–rock type relationship provides predictive power for the emplacement locations of exotic CO_2_-rich magmas and, by inference, their associated economic deposits. Our attention must now turn to elucidating the geologic drivers behind this relationship, as large gaps exist in our understanding of the relative roles of the asthenosphere and the metasomatized mantle lithosphere in the petrogenesis of CO_2_-rich magmas. Identifying these drivers will, in turn, inform future studies in determining whether this lithospheric thickness–magma type relationship has changed through geologic time—a question of particular importance given that some of the largest REE deposits (for example, Bayan Obo, Mountain Pass and Mount Weld) formed >200 Ma (ref. ^63^).

## Methods

### Geochemical compilation of CO_2_-rich magmas

For this study, we assembled a comprehensive compilation of the location, geochemistry and geochronology of a variety of CO_2_-rich intraplate continental igneous rocks (Supplementary Data 1) postulated to be derived at least in part from the lithospheric mantle^11,23,27,28,43,49,72^. These include kimberlites, olivine lamproites, ultramafic lamprophyres, melilitites, melilititic nephelinites, nephelinites, basanites and carbonatites.

Kimberlite and ultramafic lamprophyre data were taken from Giuliani et al.^23^, the latter of which was supplemented with additional samples from published literature. The kimberlite dataset includes only archetypal (group I) kimberlites. Orangeitic (group II) kimberlites, although CO_2_ rich, are more similar mineralogically and geochemically to olivine lamproites than they are to archetypal kimberlites and are thus classified as carbonate-rich olivine lamproites according to the definition of Pearson et al.^73^. For our olivine lamproite dataset, we compile these carbonate-rich as well as carbonate-poor olivine lamproites from the datasets of Giuliani et al.^23^ and Sarkar et al.^72,74^, which we append with samples from other published studies. Magmatic carbonatite localities are from Schmidt et al.^49^. We include in our carbonatite compilation both monogenetic and polyphase intrusions, as the majority of these are emplaced along craton margins^11^ and thus document similar effects of lithospheric thickness on their petrogenesis, irrespective of carbonatite subtype. Carbonatite-hosted REE deposit data are from Orris et al.^75^. We used precompiled melilitite^76^ and nephelinite^77^ files from GEOROC (https://georoc.eu/) downloaded in June 2024. On the basis of the definition of Le Bas^78^ and Le Maitre et al.^79^, we reclassified these samples into melilitites (normative larnite >10%), melilititic nephelinites (normative larnite <10%) and nephelinites, the latter of which includes both melanephelinites (normative nepheline <20% and normative albite <5%) and bona fide nephelinites (normative nepheline >20%; Supplementary Fig. 1). Basanite data are from the Neogene dataset of Ball et al.^7^. Only those basanite samples with normative nepheline <20% and normative albite >5% were included in our database in accordance with the basanite classification of Le Bas^78^ (Supplementary Fig. 1). Finally, we use alkali and subalkaline basalts from the Ball et al.^7^ dataset to represent endmember asthenospheric melts, as the compositions of these rock types are similar to ocean island basalts sourced from the sublithospheric convecting mantle^80^. For all datasets, any missing location and age information were added to samples by referencing the original publication. Only mafic silicate magmas (MgO >5 wt%) were considered to exclude highly evolved samples.

In their study of the temporal distribution of kimberlites and carbonatites, Liu et al.^81^ found that throughout Earth’s history, secular mantle cooling and a transition to deep subduction led to an overall increase in CO_2_-rich magmatism over time. Superimposed on this first-order trend are peaks in the frequency of kimberlites and carbonatites that coincide with supercontinent break-up events. Therefore, to remove the influence of long-term thermo-tectonic changes in the lithospheric mantle, only samples emplaced after the break-up of Pangea (~200 Ma), which represents the last major plate reorganization event, were included in our datasets^11^. As lithospheric thickness estimates are derived from modern shear-wave velocity anomalies, this age cut-off also minimizes the likelihood that changes in lithospheric thickness occurred after magma emplacement.

Because large location–geochemistry datasets are susceptible to uneven data coverage, we then grouped samples from each dataset into 1° × 1° bins to avoid bias resulting from oversampled regions. This binning was performed using the PyGMT Python wrapper for GMT^70,71^, which, for each spatial bin, produced a median for each parameter in our global dataset.

### Tomographic model

In this study, we use the SL2013 global tomographic model of Schaeffer and Lebedev^17^. SL2013 is built from >500,000 vertical-component waveform fits of seismograms containing both surface and body waves, with a period range from <15 s to >300 s and resolving power from the crust to the bottom of the upper mantle. Within this model, shear-wave velocity anomalies (dVs) are parametrized for 18 depths, including the 110-km depth knot. We combined our spatially averaged geochemical datasets with the 110-km depth slice of the SL2013 model (Fig. 1), as cratonic lithosphere, being cold and thick, is identifiable at 100−150 km depths as regions of high dVs (>4%).

### Lithospheric thickness from seismic thermography

To estimate lithospheric thicknesses for the different types of intraplate continental CO_2_-rich magmas, we integrated the azimuthally anisotropic, vertically-polarized shear-wave velocity tomography model of Schaeffer and Lebedev^17^ and radial anisotropy information from Lavoué et al.^82^ to calculate both Rayleigh and Love dispersion curves at every location in our spatially averaged dataset. For each magma sample location, we then perform a thermodynamic, gradient-search inversion for equilibrium geotherms and lithospheric thicknesses that best fits the dispersion curves calculated from the topography^18,21,83^. Azimuthal anisotropy was inverted for and isolated from the isotropic-average velocities by the tomographic models. Radial anisotropy was inverted for in our thermodynamic inversions to isolate the isotropic-average elastic properties of the rock at depth relating to its temperature.

In these inversions, we assume a peridotitic mantle that can be defined in the SiO_2_–Al_2_O_3_–FeO–MgO–CaO system, as the seismic wave speed anomalies in the upper mantle caused by chemical composition are much smaller than those caused by temperature^83^. Specifically, we set the FeO and Al_2_O_3_ contents according to tectonic type (that is, cratons, platforms and tectonic continents). Weight percentages of MgO and CaO are computed from Al_2_O_3_ content on the basis of correlations observed globally in xenolith samples and peridotite massifs^84^. SiO_2_ is then added to the system to attain a major element oxide total of 100%. For each bulk composition, the stable mineral assemblage is calculated at each pressure and temperature using thermodynamic databases^85,86^ in conjunction with Perple_X^87,88^. Although important in the petrogenesis of low-degree CO_2_-rich magmas, amphibole and phlogopite are not included in the mantle mineral thermodynamic database of Stixrude and Lithgow-Bertelloni^86^ and are therefore not considered in our modelling. The exclusion of amphibole and phlogopite, however, should not substantially affect our lithospheric thickness estimates, as these phases are highly localized in the lithospheric mantle—commonly occurring within thin, centimetre-scale veins—and are volumetrically insignificant (<5 vol%) in average on- and off-craton peridotite^89^.

With mineral assemblages computed and their elastic properties known, seismic velocity and density profiles are calculated accordingly. Rayleigh and Love dispersion curves from the global tomographic models^17,82^ are compared with those derived from our thermodynamic modelling. Iterations of the previous steps—performed by repeatedly perturbing the temperature profile—are conducted until the misfit between dispersion curves calculated from thermodynamic modelling and those derived from global tomographic models is lower than 0.05%. Temperature profiles obtained from this method correspond well with temperature-depth estimates derived from mantle xenolith thermobarometry^21^, thus confirming the applicability of this method in the calculation of lithospheric thickness.

We define the LAB as the bottom of the mechanical lithosphere, which is characterized by the temperature at which convection commences (Extended Data Fig. 1). We take this temperature to be 1,290 °C (refs. ^21,83^). Between the mechanical lithosphere and the fully convecting asthenosphere, there is a buffer layer, also referred to as the thermal or rheological boundary layer^83^, where heat transfer is via a mixture of conduction and convection. Within this layer, temperature increases from that of the bottom of the mechanical lithosphere to that of the fully convecting asthenosphere.

Furthermore, we note that the choice of temperature of the mechanical LAB is somewhat arbitrary, with some authors using a temperature as low as 1,175 °C (ref. ^90^). Further tests show that if we choose an LAB temperature of 1,175 °C, for example, the temperature profile remains a similar shape but the resulting LAB is ~10–30 km shallower, with greater differences for thicker lithosphere (Supplementary Fig. 2). Therefore, to account for uncertainties in our lithospheric thickness estimates (see Xu et al.^21^ for a full discussion of the uncertainties in these calculations), we assign a 10% uncertainty to each estimate following the general uncertainty level in LAB depths from the global study of Xu et al.^21^ (Extended Data Fig. 2).

Finally, because the isotropic and anisotropic shear-wave velocity models have a lateral resolution of a few hundred kilometres, the LAB depths reported in this study approximate an average value for an area several hundreds-of-kilometres wide. As a result, small-scale lateral variations in LAB depth can be underestimated in their amplitude. This is the case for the localized thick lithosphere^64–66^ of the Colorado Plateau, western North America, which has a lateral extent of up to 150 km; we therefore exclude lithospheric thickness estimates for samples from this region, which record seemingly much thinner lithosphere (~75 km). We also discarded lithospheric thickness estimates for the Tibetan Plateau, Central Asia, as this region is underplated by the downgoing eclogitized Indian lithosphere^91^. In this setting, temperature does not always increase with depth, which, coupled with the presence of non-peridotitic mantle, contradicts the assumption of equilibrium geotherms made in our thermodynamic inversions.

## Online content

Any methods, additional references, Nature Portfolio reporting summaries, source data, extended data, supplementary information, acknowledgements, peer review information; details of author contributions and competing interests; and statements of data and code availability are available at 10.1038/s41561-026-01990-7.

## Supplementary information

Supplementary Information Supplementary Figs. 1 and 2.

Supplementary Data 1 Location, geochemistry and age information for CO_2_-rich intraplate continental igneous rock samples used in this study as well as lithospheric thickness estimates for the spatially averaged magma type dataset, where the mechanical LAB is defined as either the 1,290 °C or 1,175 °C isotherm.

## Data Availability

All geophysical data used in this study can be acquired via the references provided (for example, refs. ^17,67,82^). Topographic data used in this study are from SRTM15+^68^ and available via figshare at 10.6084/m9.figshare.7979780.v2 (ref. ^69^). All published location–geochemistry data for CO_2_-rich magmas used in this study are provided with reference details in Supplementary Data 1. The kimberlite dataset is from Giuliani et al.^23^; the ultramafic lamprophyre dataset is modified from Giuliani et al.^23^; the olivine lamproite dataset is modified from Giuliani et al.^23^, Sarkar et al.^72^ and Sarkar et al.^74^ (10.60520/IEDA/113512); the carbonatite dataset is modified from Schmidt et al.^49^ with REE deposit information from Orris et al.^75^ (10.5066/F7DR2TN4); and the basanite and basalt dataset is modified from Ball et al.^7^. We used precompiled melilitite^76^ (10.25625/2JETOA) and nephelinite^77^ (10.25625/2JETOA) files, part of the GEOROC Compilation: Rock Types database, that were downloaded from GEOROC (https://georoc.eu/) in June 2024. All new data (lithospheric thickness estimates calculated for our spatially averaged magma type dataset) presented in this paper are reported in Supplementary Data 1 and are available via figshare at 10.6084/m9.figshare.29414012 (ref. ^92^).

## Extended data

**Extended Data Table 1:** Median lithospheric thickness estimates and associated statistics for each magma type in our spatially averaged dataset

Magma Type	Count	Median	Std. Dev.	Min.	Max.	Quartile 1	Quartile 3
Subalkaline basalt	297	72	17	36	129	62	81
Alkali basalt	358	74	16	38	135	65	84
Basanite	312	77	16	42	192	68	87
Nephelinite	117	89	28	54	175	77	108
Melilititic nephelinite	97	95	28	56	218	80	114
Melilitite	42	95	29	66	218	84	110
Ultramafic lamprophyre	22	102	22	76	168	96	117
Olivine lamproite	35	180	46	64	247	153	216
Kimberlite	52	206	44	112	274	167	236
Carbonatite	111	114	44	51	263	95	140
Carbonatite-hosted REE deposit	31	127	43	59	245	108	147

Count refers to the number of samples of each magma type in our spatially averaged dataset. The median lithospheric thickness estimate (km), the standard deviation of lithospheric thickness estimates (km), and the minimum and maximum lithospheric thickness estimates (km) for each magma type are reported. Quartile 1 (km) and quartile 3 (km) bracket the middle 50% range of lithospheric thickness estimates for each magma type.

**Extended Data Table 2:** Median Al_2_O_3_ (wt.%), Dy/Yb, La/Sm, and K_2_O/Na_2_O and associated sample size (count) for each magma type in our spatially averaged geochemical dataset

Magma Type	Median Al_2_O_3_	Count	Median Dy/Yb	Count	Median La/Sm	Count	Median K_2_O/Na_2_O	Count
Subalkaline basalt	14.54	233	2.44	156	3.97	188	0.30	233
Alkali basalt	14.30	302	2.79	203	4.74	248	0.40	302
Basanite	13.75	263	3.17	174	5.75	216	0.43	263
Nephelinite	11.60	102	3.33	60	6.35	77	0.44	102
Melilititic nephelinite	10.12	94	3.38	52	7.20	70	0.51	94
Melilitite	9.27	41	3.58	27	7.39	28	0.66	41
Ultramafic lamprophyre	6.84	21	3.86	9	8.33	11	1.84	21
Olivine lamproite	4.70	35	4.15	24	11.56	26	8.41	33
Kimberlite	2.77	54	4.43	38	10.19	42	4.90	52

## Code availability

The PyGMT Python wrapper for GMT^70,71^ was used to create figures and generate spatially averaged datasets. Code used in this study to calculate lithospheric thickness estimates is based on the LitMod3D software^83^, which is available via GitHub at https://github.com/javfurchu/LitMod3D_V3.1.
