# Supplementary material for: The global distribution of CO2-rich magmas is determined by lithospheric thickness
Source: Nat Geosci. 2026 May 22;19(6):732–8. doi: 10.1038/s41561-026-01990-7 (PMC13259920; doi:10.1038/s41561-026-01990-7)
Supplement: Supplementary file 1 — Supplementary Figs. 1 and 2. [file 41561_2026_1990_MOESM1_ESM.pdf]

nature geoscience

Supplementary information https://doi.org/10.1038/s41561-026-01990-7

The global distribution of CO_2-rich magmas is determined by lithospheric thickness

In the format provided by the authors and unedited

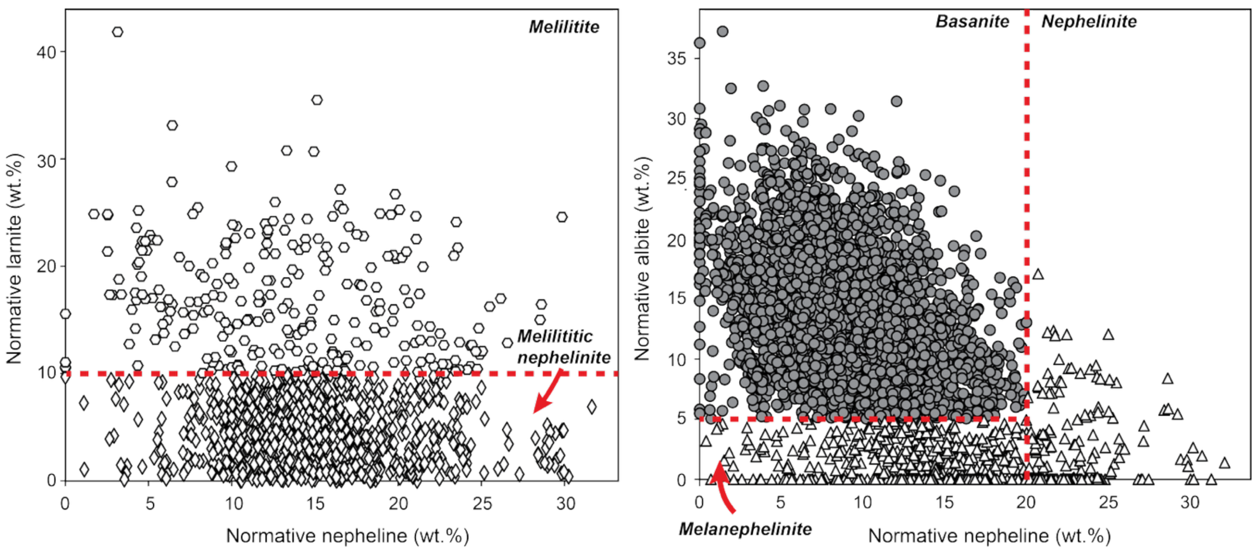

Supplementary Fig. 1. Discrimination diagrams for carbonated sodic silicate magmas. Basanite data are from Ball et al.[7] and melilitite through nephelinite data are from the GEOROC (https://georoc.eu/) melilitite[76] and nephelinite[77] precompiled files. Hexagon symbols are melilitites, diamonds are melilititic nephelinites, triangles are nephelinites (which includes both melanephelinites and *bona fide* nephelinites), and grey circles are basanites.

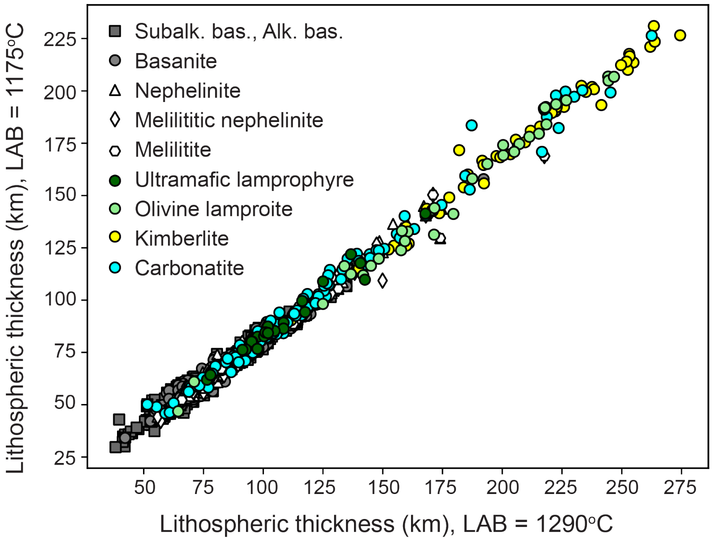

Supplementary Fig. 2. Comparison of lithospheric thickness estimates for our spatially averaged magma-type dataset derived using LAB temperatures of 1290°C and 1175°C. Subalk. bas. = subalkaline basalt; Alk. bas. = alkali basalt. Data sources for samples are listed in the Online Methods and Supplementary Data 1.
